# 5-Chloro-2-hydroxy­benzene-1,3-dicarb­aldehyde

**DOI:** 10.1107/S1600536808020217

**Published:** 2008-07-09

**Authors:** Jue-Chao Jiang, Gang Wang, Wei You, Wei Huang

**Affiliations:** a State Key Laboratory of Coordination Chemistry, Coordination Chemistry Institute, School of Chemistry and Chemical Engineering, Nanjing University, Nanjing, 210093, People’s Republic of China; b College of Sciences, Nanjing University of Technology, Nanjing, 210009, People’s Republic of China

## Abstract

In the crystal structure of the title compound, C_8_H_5_ClO_3_, both formyl groups are in the plane of the chloro­phenyl unit and the mol­ecule is stabilized by intra­molecular O—H⋯O hydrogen bonding. The mol­ecules are connected *via* inter­molecular O—H⋯O hydrogen bonding into chains and are stacked into columns with a centroid–centroid distance between adjacent aromatic rings of 3.914 (2) Å.

## Related literature

For related compounds, see: Huang *et al.* (2000 ▶, 2006 ▶); Chu *et al.* (2005 ▶); Chu & Huang (2006 ▶).
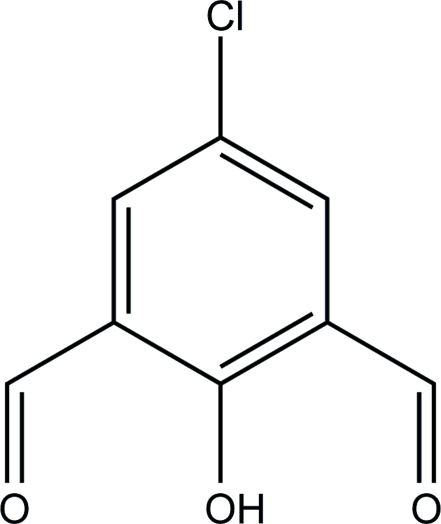


## Experimental

### 

#### Crystal data

C_8_H_5_ClO_3_
*M* *_r_* = 184.57
Monoclinic, 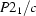

*a* = 7.5554 (15) Å
*b* = 3.9144 (8) Å
*c* = 25.676 (5) Å
β = 97.921 (3)°
*V* = 752.1 (3) Å^3^
*Z* = 4
Mo *K*α radiation
μ = 0.46 mm^−1^
*T* = 291 (2) K
0.20 × 0.18 × 0.16 mm

#### Data collection

Bruker SMART CCD area-detector diffractometer
Absorption correction: multi-scan (*SADABS*; Bruker, 2000 ▶) *T*_min_ = 0.913, *T*_max_ = 0.930
3517 measured reflections
1353 independent reflections
1070 reflections with *I* > 2σ(*I*)
*R*_int_ = 0.076

#### Refinement

*R*[*F* ^2^ > 2σ(*F* ^2^)] = 0.035
*wR*(*F* ^2^) = 0.091
*S* = 0.97
1353 reflections
125 parameters
H atoms treated by a mixture of independent and constrained refinement
Δρ_max_ = 0.18 e Å^−3^
Δρ_min_ = −0.27 e Å^−3^

### 

Data collection: *SMART* (Bruker, 2000 ▶); cell refinement: *SAINT* (Bruker, 2000 ▶); data reduction: *SAINT*; program(s) used to solve structure: *SHELXTL* (Sheldrick, 2008 ▶); program(s) used to refine structure: *SHELXTL*; molecular graphics: *SHELXTL*; software used to prepare material for publication: *SHELXTL*.

## Supplementary Material

Crystal structure: contains datablocks global, I. DOI: 10.1107/S1600536808020217/nc2109sup1.cif

Structure factors: contains datablocks I. DOI: 10.1107/S1600536808020217/nc2109Isup2.hkl

Additional supplementary materials:  crystallographic information; 3D view; checkCIF report

## Figures and Tables

**Table 1:** Hydrogen-bond geometry (Å, °)

*D*—H⋯*A*	*D*—H	H⋯*A*	*D*⋯*A*	*D*—H⋯*A*
O1—H1⋯O1^i^	0.82	2.48	2.9581 (19)	118
O1—H1⋯O3	0.82	1.90	2.6204 (18)	146

Symmetry code: (i) 
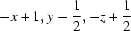
.

**Fig. 1.**
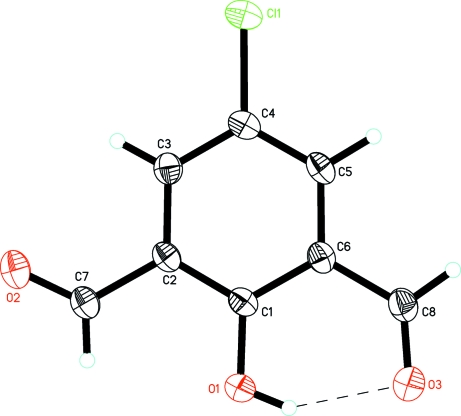
An ORTEP drawing of the title compound with the atom-numbering scheme. Displacement ellipsoids are drawn at the 30% probability level and intramolecular hydrogen bonds is shown as dashed lines.

**Fig. 2.**
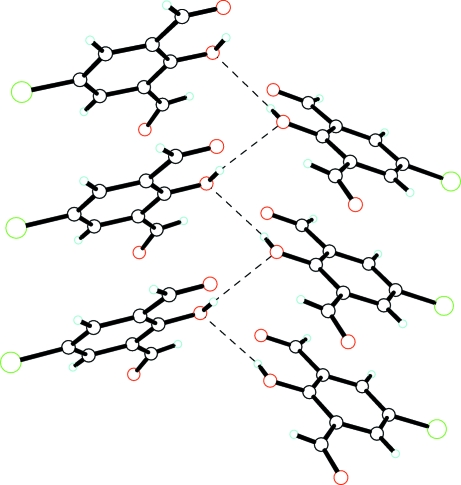
A perspective view of the one-dimensional hydrogen-bonded chain of the title compound.

**Fig. 3.**
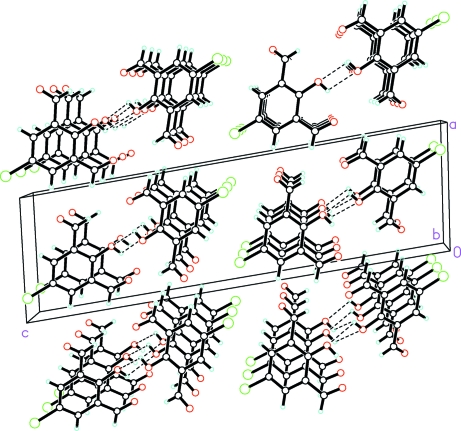
Crystal structure of the title compound with view along the a-axis (intermolecular O-H···O hydrogen bonding is shown as dashed lines).

## supplementary crystallographic information

### Comment

Hydroxyisophthalaldehyde and its derivatives are an important class of intermediates used in synthesizing macrocyclic compounds. In recent years, a continuing attention has been drawn to them and their metal complexes (Huang *et al.*, 2006). In this paper, we report the X-ray single-crystal structure of 2,6-diformyl-4-chlorophenol prepared from 4-chloro-2,6-bis(hydroxymethyl)phenol.

The molecule of the title compound is essentially planar and all structural parameters (Fig. 1) are in good agreement with those found in similar compounds (Chu *et al.*, 2005; Chu & Huang, 2006). There is one weak intramolecular O-H···O hydrogen bond between the hydroxyl group at O1 and the carbonyl group O3.

In the crystal structure of the title compound the molecules are connected into chains by intermolecular O-H···O hydrogen bonding (Fig. 2 and Table 1). The molecules are stacked into columns in the direction of the crystallographic a-axis in order that π–π stacking interactions are maximized. The dihedral angle between two adjacent rings amount to 63.5 (2)° and the centroid-centroid separation is 3.914 (2) Å (Fig. 3).

### Experimental

4-Chloro-2,6-diformylphenol was prepared by an improved oxidation method using activated manganese (IV) dioxide (Huang *et al.*, 2000) from 4-chloro-2,6-bis(hydroxymethyl)phenol (Chu *et al.*, 2005). Single crystals suitable for X-ray diffraction measurement were grown from a chloroform solution by slow evaporation of the solvent at room temperature.

### Refinement

The C-H H atoms were located in difference map and were refined with varying coordinates isotropic. The O-H H atom was placed with idealized geometry allowed to rotata but not to tip O—H = 0.82 Å) and was refined using a riding model with *U*_iso_(H) = 1.5*U*_eq_(O).

### Crystal data

**Table:**

C_8_H_5_ClO_3_	*F*_000_ = 376
*M**_r_* = 184.57	*D*_x_ = 1.630 Mg m^−^^3^
Monoclinic, *P*2_1_/*c*	Mo *K*α radiation λ = 0.71073 Å
Hall symbol: -P 2ybc	Cell parameters from 1370 reflections
*a* = 7.5554 (15) Å	θ = 2.7–28.1º
*b* = 3.9144 (8) Å	µ = 0.46 mm^−^^1^
*c* = 25.676 (5) Å	*T* = 291 (2) K
β = 97.921 (3)º	Block, yellow
*V* = 752.1 (3) Å^3^	0.20 × 0.18 × 0.16 mm
*Z* = 4	

### Data collection

**Table:**

Bruker SMART CCD area-detector diffractometer	1353 independent reflections
Radiation source: fine-focus sealed tube	1070 reflections with *I* > 2σ(*I*)
Monochromator: graphite	*R*_int_ = 0.076
*T* = 291(2) K	θ_max_ = 25.2º
φ and ω scans	θ_min_ = 2.7º
Absorption correction: multi-scan(SADABS; Bruker, 2000)	*h* = −9→8
*T*_min_ = 0.913, *T*_max_ = 0.930	*k* = −4→4
3517 measured reflections	*l* = −30→28

### Refinement

**Table:**

Refinement on *F*^2^	Secondary atom site location: difference Fourier map
Least-squares matrix: full	Hydrogen site location: inferred from neighbouring sites
*R*[*F*^2^ > 2σ(*F*^2^)] = 0.035	H atoms treated by a mixture of independent and constrained refinement
*wR*(*F*^2^) = 0.091	*w* = 1/[σ^2^(*F*_o_^2^) + (0.0457*P*)^2^] where *P* = (*F*_o_^2^ + 2*F*_c_^2^)/3
*S* = 0.97	(Δ/σ)_max_ = 0.001
1353 reflections	Δρ_max_ = 0.19 e Å^−^^3^
125 parameters	Δρ_min_ = −0.27 e Å^−^^3^
Primary atom site location: structure-invariant direct methods	Extinction correction: none

### Special details

**Table:**

Experimental. The structure was solved by direct methods (Bruker, 2000) and successive difference Fourier syntheses.
Geometry. All e.s.d.'s (except the e.s.d. in the dihedral angle between two l.s. planes) are estimated using the full covariance matrix. The cell e.s.d.'s are taken into account individually in the estimation of e.s.d.'s in distances, angles and torsion angles; correlations between e.s.d.'s in cell parameters are only used when they are defined by crystal symmetry. An approximate (isotropic) treatment of cell e.s.d.'s is used for estimating e.s.d.'s involving l.s. planes.
Refinement. Refinement of *F*^2^ against ALL reflections. The weighted *R*-factor *wR* and goodness of fit *S* are based on *F*^2^, conventional *R*-factors *R* are based on *F*, with *F* set to zero for negative *F*^2^. The threshold expression of *F*^2^ > σ(*F*^2^) is used only for calculating *R*-factors(gt) *etc*. and is not relevant to the choice of reflections for refinement. *R*-factors based on *F*^2^ are statistically about twice as large as those based on *F*, and *R*- factors based on ALL data will be even larger.

### Fractional atomic coordinates and isotropic or equivalent isotropic displacement parameters (Å^2^)

**Table:**

	*x*	*y*	*z*	*U*_iso_*/*U*_eq_	
C1	0.4171 (2)	0.4316 (4)	0.33617 (6)	0.0342 (4)	
C2	0.5238 (2)	0.6213 (5)	0.37437 (7)	0.0355 (4)	
C3	0.4574 (2)	0.7115 (5)	0.42026 (8)	0.0384 (4)	
C4	0.2876 (2)	0.6140 (5)	0.42836 (7)	0.0382 (4)	
C5	0.1802 (2)	0.4303 (5)	0.39068 (7)	0.0372 (4)	
C6	0.2425 (2)	0.3385 (5)	0.34400 (7)	0.0348 (4)	
C7	0.7038 (3)	0.7330 (5)	0.36545 (9)	0.0447 (5)	
C8	0.1285 (3)	0.1496 (5)	0.30341 (8)	0.0426 (5)	
Cl1	0.20868 (7)	0.73166 (14)	0.486064 (19)	0.0550 (2)	
H3	0.532 (3)	0.831 (5)	0.4454 (7)	0.047 (5)*	
H5	0.064 (3)	0.358 (5)	0.3977 (8)	0.052 (5)*	
H7	0.747 (3)	0.653 (5)	0.3360 (8)	0.052 (6)*	
H8	0.015 (2)	0.099 (5)	0.3119 (7)	0.044 (5)*	
O1	0.48613 (16)	0.3399 (3)	0.29244 (5)	0.0467 (4)	
H1	0.4106	0.2351	0.2726	0.070*	
O2	0.79414 (18)	0.9339 (4)	0.39307 (6)	0.0606 (4)	
O3	0.17345 (17)	0.0628 (4)	0.26168 (5)	0.0544 (4)	

### Atomic displacement parameters (Å^2^)

**Table:**

	*U*^11^	*U*^22^	*U*^33^	*U*^12^	*U*^13^	*U*^23^
C1	0.0313 (9)	0.0364 (10)	0.0359 (9)	0.0027 (8)	0.0079 (7)	0.0064 (8)
C2	0.0293 (9)	0.0375 (10)	0.0404 (10)	0.0016 (8)	0.0068 (7)	0.0059 (8)
C3	0.0349 (10)	0.0375 (11)	0.0424 (11)	−0.0001 (8)	0.0034 (8)	0.0017 (8)
C4	0.0372 (10)	0.0403 (10)	0.0388 (10)	0.0048 (8)	0.0110 (8)	0.0052 (8)
C5	0.0266 (9)	0.0412 (11)	0.0447 (10)	0.0026 (8)	0.0078 (8)	0.0096 (8)
C6	0.0295 (10)	0.0352 (10)	0.0392 (10)	0.0020 (7)	0.0026 (7)	0.0074 (8)
C7	0.0320 (11)	0.0524 (13)	0.0504 (12)	−0.0027 (9)	0.0083 (9)	−0.0016 (10)
C8	0.0329 (11)	0.0487 (12)	0.0456 (11)	−0.0010 (9)	0.0036 (8)	0.0062 (9)
Cl1	0.0548 (4)	0.0668 (4)	0.0475 (3)	−0.0021 (2)	0.0221 (2)	−0.0052 (2)
O1	0.0400 (7)	0.0626 (9)	0.0393 (7)	−0.0074 (6)	0.0120 (5)	−0.0060 (6)
O2	0.0404 (8)	0.0732 (10)	0.0689 (9)	−0.0158 (8)	0.0099 (7)	−0.0082 (8)
O3	0.0454 (8)	0.0680 (10)	0.0488 (8)	−0.0076 (7)	0.0036 (6)	−0.0092 (7)

### Geometric parameters (Å, °)

**Table:**

C1—O1	1.3500 (19)	C5—C6	1.394 (2)
C1—C2	1.395 (2)	C5—H5	0.96 (2)
C1—C6	1.409 (2)	C6—C8	1.459 (3)
C2—C3	1.388 (2)	C7—O2	1.206 (2)
C2—C7	1.476 (2)	C7—H7	0.92 (2)
C3—C4	1.382 (2)	C8—O3	1.216 (2)
C3—H3	0.926 (19)	C8—H8	0.938 (18)
C4—C5	1.377 (3)	O1—H1	0.8200
C4—Cl1	1.7337 (17)		
			
O1—C1—C2	118.32 (15)	C4—C5—H5	119.1 (12)
O1—C1—C6	121.75 (15)	C6—C5—H5	120.6 (12)
C2—C1—C6	119.93 (15)	C5—C6—C1	119.33 (16)
C3—C2—C1	119.40 (16)	C5—C6—C8	120.57 (16)
C3—C2—C7	120.30 (17)	C1—C6—C8	120.10 (16)
C1—C2—C7	120.27 (16)	O2—C7—C2	124.11 (19)
C4—C3—C2	120.61 (18)	O2—C7—H7	118.0 (13)
C4—C3—H3	121.7 (12)	C2—C7—H7	117.8 (13)
C2—C3—H3	117.7 (12)	O3—C8—C6	124.18 (18)
C5—C4—C3	120.54 (16)	O3—C8—H8	121.5 (11)
C5—C4—Cl1	120.03 (13)	C6—C8—H8	114.3 (11)
C3—C4—Cl1	119.42 (15)	C1—O1—H1	109.5
C4—C5—C6	120.17 (16)		

### Hydrogen-bond geometry (Å, °)

**Table:**

*D*—H···*A*	*D*—H	H···*A*	*D*···*A*	*D*—H···*A*
O1—H1···O1^i^	0.82	2.48	2.9581 (19)	118
O1—H1···O3	0.82	1.90	2.6204 (18)	146

Symmetry codes: (i) −*x*+1, *y*−1/2, −*z*+1/2.
